# Supplementary material for: Effects of Trichoderma harzianum combined with Phanerochaete chrysosporium on lignin degradation and humification during chicken manure and rice husk composting
Source: Front Microbiol. 2025 Feb 28;16:1515931. doi: 10.3389/fmicb.2025.1515931 (PMC11906335; doi:10.3389/fmicb.2025.1515931)
Supplement: Supplementary file 1 [file Data_Sheet_1.zip › 6.Function/All/FunGuild/l2/NMDS.pdf]

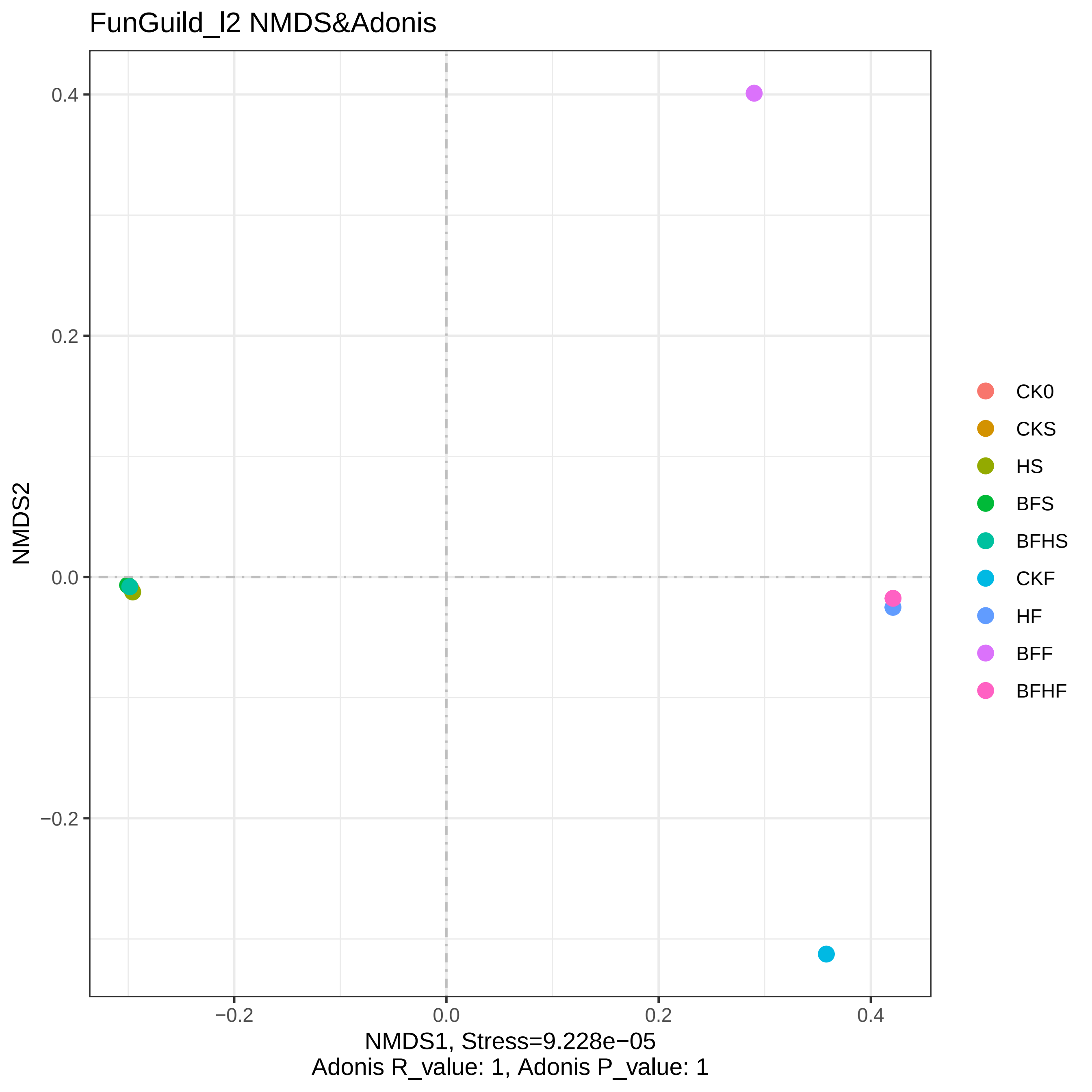
FunGuild_l2 NMDS&Adonis
0.4
0.2
0.0
−0.2
NMDS2
−0.2
0.0
0.2
0.4
NMDS1, Stress=9.228e−05
Adonis R_value: 1, Adonis P_value: 1
CK0
CKS
HS
BFS
BFHS
CKF
HF
BFF
BFHF
